# Reviewed article: Nunes P, Ditterich RG, Colussi CF, Godoi H, Mello ALSF. Management performance in public oral health actions and services: a cross-sectional study in the Serra Catarinense region, Brazil, 2022. Epidemiol Serv Saude. 2025;33:e20240184

**DOI:** 10.1590/S2237-96222025v34e20240184.b

**Published:** 2025-06-16

**Authors:** Marcelo Coelho Goiato

**Affiliations:** Universidade Estadual Paulista (UNESP), São Paulo- SP.

**Table:** 

Rating	Excellent	Good	Average	Below Poor
Interest		✓		
Quality		✓		
Originality		✓		
Overall	✓	✓		

**Table:** 

Questionnaire	Yes	No	Not applicable
Does the manuscript contain new and significant information to justify publication?	✓		
Does the Abstract (Summary) clearly and accurately describe the content of the article?	✓		
Is the problem significant and concisely stated?		✓	
Are the methods described comprehensively?	✓		
Are the interpretations and conclusions justified by the results?	✓		
Is adequate reference made to other work in the field?	✓		

## Manuscript Structure

Length of article is: Adequate

Number of tables is: Adequate

Number of figures is: Adequate

Please state any conflict(s) of interest that you have in relation to the review of this paper (state “none” if this is not applicable): None

## Comments

O manuscrito é bastante interessante tem como objetivo avaliar o desempenho da gestão nas ações e serviços públicos de saúde bucal de gestores municipais. Julgo muito pertinente para ser divulgado. resultados bem promissores.

